# Gene Therapy With Angiotensin-(1-9) Preserves Left Ventricular Systolic Function After Myocardial Infarction

**DOI:** 10.1016/j.jacc.2016.09.946

**Published:** 2016-12-20

**Authors:** Caroline Fattah, Katrin Nather, Charlotte S. McCarroll, Maria P. Hortigon-Vinagre, Victor Zamora, Monica Flores-Munoz, Lisa McArthur, Lorena Zentilin, Mauro Giacca, Rhian M. Touyz, Godfrey L. Smith, Christopher M. Loughrey, Stuart A. Nicklin

**Affiliations:** a Institute of Cardiovascular and Medical Sciences, University of Glasgow, Glasgow, United Kingdom; b Universidad Veracruzana, Xalapa, Mexico; c International Centre for Genetic Engineering and Biotechnology, Trieste, Italy

**Keywords:** adeno-associated virus, calcium, inotropy, renin angiotensin system, Ang, angiotensin, AT_2_R, angiotensin type 2 receptor, CO, cardiac output, FS, fractional shortening, LV, left ventricular, MI, myocardial infarction, RAAS, renin-angiotensin-aldosterone system, SERCA, sarcoplasmic endoreticulum calcium adenosine triphosphatase

## Abstract

**Background:**

Angiotensin-(1-9) [Ang-(1-9)] is a novel peptide of the counter-regulatory axis of the renin-angiotensin-aldosterone system previously demonstrated to have therapeutic potential in hypertensive cardiomyopathy when administered via osmotic mini-pump. Here, we investigate whether gene transfer of Ang-(1-9) is cardioprotective in a murine model of myocardial infarction (MI).

**Objectives:**

The authors evaluated effects of Ang-(1-9) gene therapy on myocardial structural and functional remodeling post-infarction.

**Methods:**

C57BL/6 mice underwent permanent left anterior descending coronary artery ligation and cardiac function was assessed using echocardiography for 8 weeks followed by a terminal measurement of left ventricular pressure volume loops. Ang-(1-9) was delivered by adeno-associated viral vector via single tail vein injection immediately following induction of MI. Direct effects of Ang-(1-9) on cardiomyocyte excitation/contraction coupling and cardiac contraction were evaluated in isolated mouse and human cardiomyocytes and in an ex vivo Langendorff-perfused whole-heart model.

**Results:**

Gene delivery of Ang-(1-9) reduced sudden cardiac death post-MI. Pressure volume measurements revealed complete restoration of end-systolic pressure, ejection fraction, end-systolic volume, and the end-diastolic pressure volume relationship by Ang-(1-9) treatment. Stroke volume and cardiac output were significantly increased versus sham. Histological analysis revealed only mild effects on cardiac hypertrophy and fibrosis, but a significant increase in scar thickness. Direct assessment of Ang-(1-9) on isolated cardiomyocytes demonstrated a positive inotropic effect via increasing calcium transient amplitude and contractility. Ang-(1-9) increased contraction in the Langendorff model through a protein kinase A–dependent mechanism.

**Conclusions:**

Our novel findings showed that Ang-(1-9) gene therapy preserved left ventricular systolic function post-MI, restoring cardiac function. Furthermore, Ang-(1-9) directly affected cardiomyocyte calcium handling through a protein kinase A–dependent mechanism. These data emphasized Ang-(1-9) gene therapy as a potential new strategy in the context of MI.

## Central Illustration

**Figure:**
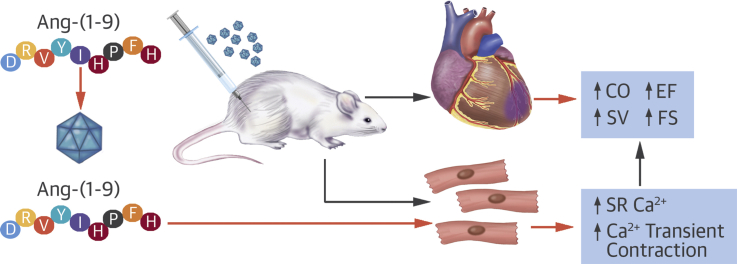

The renin-angiotensin-aldosterone system (RAAS) maintains cardiovascular homeostasis through angiotensin II (Ang II). Clinically, angiotensin-converting enzyme (ACE) inhibitors or angiotensin receptor blockers are mainstay treatments for hypertension and heart failure (HF). Following myocardial infarction (MI), RAAS inhibition stabilizes adverse cardiac remodeling and function and limits progression to HF.

A natural counter-regulatory axis of the RAAS exists, centered on ACE2, an ACE homologue that metabolizes Ang II to angiotensin-(1-7) [Ang-(1-7)] 1, 2. Currently being explored therapeutically in cardiovascular diseases including HF and pulmonary hypertension, ACE2 shows promising therapeutic effects (3). Ang-(1-7) acts via the receptor Mas to block detrimental effects of Ang II and mediates direct therapeutic effects in cardiovascular disease 4, 5. Ang-(1-7) is in clinical trials to treat diabetic foot ulcers and cancer 6, 7, emphasizing translational approaches targeting the counter-regulatory RAAS axis.

Less studied than Ang-(1-7), the alternative counter-regulatory RAAS peptide angiotensin-(1-9) [Ang-(1-9)] reduces adverse cardiovascular remodeling in rat models of hypertension and MI following peptide administration via osmotic mini-pump 8, 9, 10. Ang-(1-9) attenuates cardiomyocyte hypertrophy and cardiac fibrosis in hypertensive models; these effects are blocked by coadministration of the angiotensin type 2 receptor (AT_2_R) antagonist PD123,319, further supporting independent effects of Ang-(1-9) as a new counter-regulatory RAAS axis peptide 8, 11.

Assessment of RAAS peptides as therapeutics is limited by short circulatory half-life, requiring osmotic mini-pumps for sustained release in vivo in experimental models. Accordingly, alternative delivery strategies are required for clinical translation. Viral gene therapy is being pursued for HF, including clinical trials using adeno-associated virus (AAV) vector-mediated delivery of sarcoplasmic endoreticulum calcium adenosine triphosphatase 2a (SERCA2a), emphasizing safety and clinical utility (12).

Angiotensin peptides are not produced from genes, but are generated extracellularly in the circulation. Synthetic expression cassettes for Ang II, Ang-(1-7), and Ang-(1-9) have been utilized in transgenic models and in gene transfer approaches 13, 14, 15. Here, for the first time, in vivo AAV-mediated gene transfer of Ang-(1-9) via a synthetic expression cassette has been utilized to study cardiac effects in a murine model of MI.

## Methods

**Figure 1:**
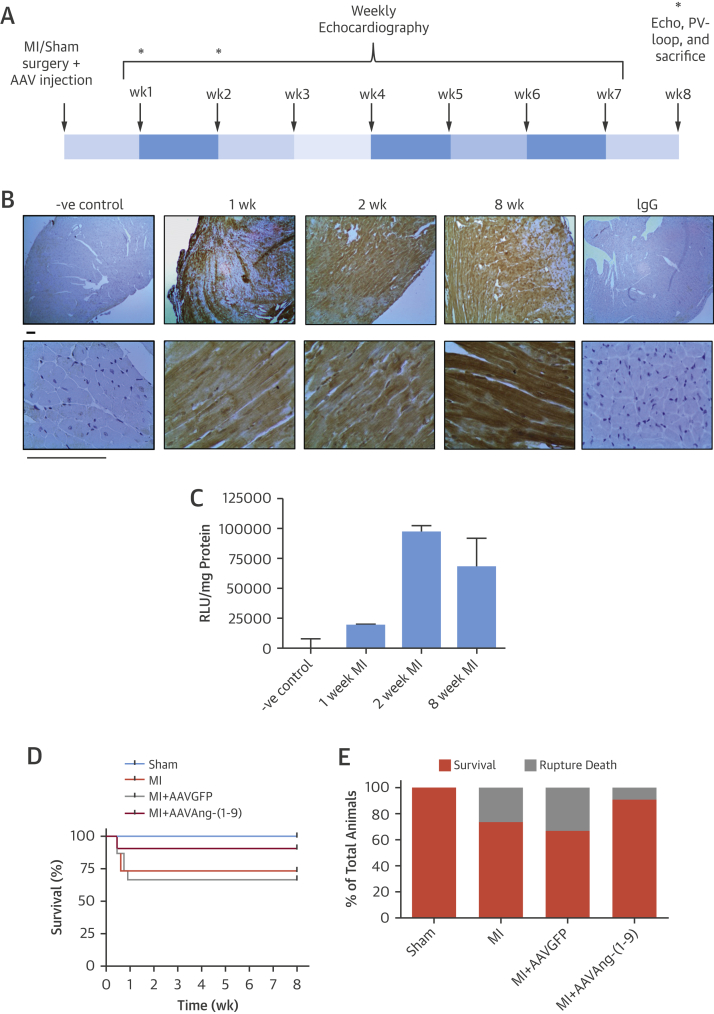
AAV Delivery **(A)** Study design. **(B)** Immunohistochemistry for enhanced GFP at 1, 2, and 8 weeks following intravenous delivery of AAVGFP. (Original magnification ×4 for upper panel and ×40 for lower panel; scale = 100 μm.) **(C)** Quantification of GFP in transduced heart lysates using GFP assay. Fluorescence normalized to negative control heart tissue basal fluorescence and total protein concentration. **(D)** Mortality for each animal group. Group sizes are n = 10, n = 15, n = 15, and n = 11 for sham, MI, MI/AAVGFP, and MI/AAV Ang-(1-9), respectively. **(E)** Percent survival and cause of mortality. AAV = adeno-associated virus; Ang-(1-9) = angiotensin-(1-9); GFP = green fluorescence protein; MI = myocardial infarction.

Detailed methods are presented in the Online Appendix. Briefly, an Ang-(1-9) expression cassette (13) was sub-cloned into plasmid adeno-associated virus-multiple cloning site (pAAV-MCS) and AAV9 vectors produced via standard protocols (16). Surgical procedures were performed in accordance with the Animals Scientific Procedures Act (1986) and approved by the University of Glasgow Animal Welfare and Ethical Review Panel and UK Home Office. For MI, the left anterior descending artery (LAD) was ligated. Sham animals had identical procedures without ligation. AAVAng-(1-9) or AAV green fluorescent protein (GFP) were delivered intravenously via tail vein following MI as described (17). Echocardiography was performed weekly (Figure 1A) and pressure volume (PV) loop measurements made. Fibrosis was assessed by Picrosirius red staining as described (8). Hypertrophy was measured by wheat germ agglutinin staining. Quantitative reverse transcription polymerase chain reaction was assessed with inventoried gene expression assays. Ventricular cardiomyocytes were isolated from adult C57BL/6 mice, loaded with Fura–4FAM, and the Fura–4FAM fluorescence ratio (340/380 nm excitation) was measured using a spinning wheel monochromator and converted to [Ca^2+^]_i_ (18). Cardiomyocytes were incubated for 15 min with 1 μmol/l Ang-(1-9), field-stimulated (1.0 Hz), and perfused with 1.8 mmol/l [Ca^2+^]_o_ HEPES superfusate containing 1 μmol/l Ang-(1-9). Calcium transients and contractility in human-induced pluripotent stem cell-derived cardiomyocytes (hiPS-CM; iCell^2^ cardiomyocytes, Cellular Dynamics International [Madison, Wisconsin, USA]) were measured in the optical platform CellOPTIQ (Clyde Biosciences Ltd, Glasgow, United Kingdom) in cells loaded with 3 μmol/l Fura-4F-AM. Calcium transients were obtained from the 360/380 ratio and contraction was assessed using a high-resolution camera coupled to CellOPTIQ. Male adult Wistar rats were sacrificed, hearts excised, and Langendorff perfused at 37°C and constant flow (10 ml/min) (19). A fluid-filled balloon was inserted into the left ventricle and connected to a solid-state pressure transducer. Hearts were paced and perfused with 1 μmol/l Ang-(1-9).

### Statistical analysis

Data are represented as mean ± SE of the mean (SEM). Paired Student *t* test for direct comparisons and 1-way analysis of variance with Tukey’s post-test for multiple comparison were performed. Echocardiography was analyzed using repeated measures analysis of variance with Tukey’s post-test. Statistical significance was demonstrated with a p < 0.05.

## Results

Previously, tail vein delivery of 1 × 10^11^ viral genomes (vg) AAV9 demonstrated robust cardiac transduction (17). To confirm this, AAVGFP-mediated transduction was assessed at 1, 2, and 8 weeks following LAD ligation (Figure 1A). High enhanced GFP expression was observed throughout the myocardium at all time points (Figure 1B). Quantification of enhanced GFP expression in cardiac lysates revealed enhanced GFP expression was detectable at 1 week and increased at 4 and 8 weeks (Figure 1C). Next, animals were subjected to sham procedure, MI, MI/AAVGFP, or MI/AAVAng-(1-9) to assess effects on cardiac function and remodeling. MI in presence or absence of AAVGFP produced higher mortality than sham in the acute recovery phase due to cardiac rupture (sham: 100% survival; MI: 73% survival; MI/AAVGFP: 67% survival) (Figures 1D and 1E). Delivery of AAVAng-(1-9) increased survival to 91% in MI-induced animals.

### Assessment of cardiac function

**Figure 2:**
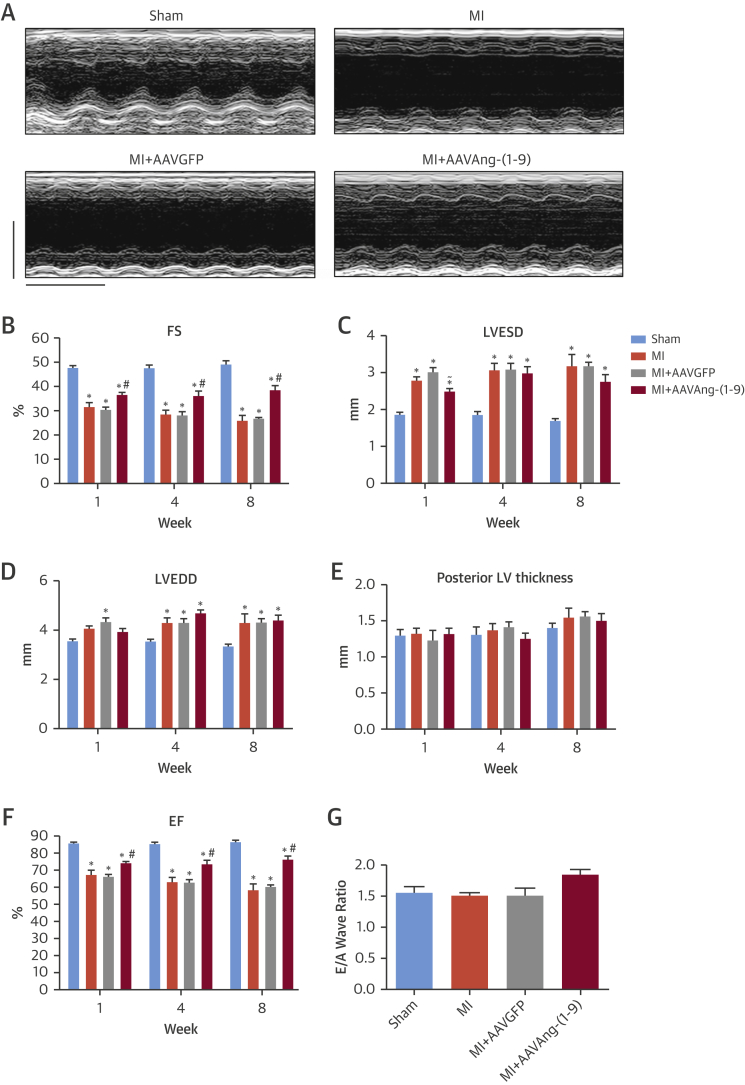
Cardiac Function **(A)** Eight-week M-mode images (scale = 5 mm and 1 s). Effect of AAVAng-(1-9) varied by parameter: **(B)** Serial FS; **(C)** LVESD; **(D)** LVEDD; **(E)** posterior LV thickness; and **(F)** EF. *p <0.05 versus sham; ^#^p <0.05 versus MI and MI/AAVGFP; ^∼^p <0.05 MI/AAVGFP versus MI/AAVAng-(1-9). **(G)** Average E/A ratio measurements (n = 6 per group). Data presented as mean ± SEM. A = after wave; E = early wave; EF = ejection fraction; FS = fractional shortening; LV = left ventricular; LVEDD = left ventricular end diastolic dimension; LVESD = left ventricular end systolic dimension; other abbreviations as in Figure 1.

Serial echocardiography was performed (Figure 2A) and a significant reduction in fractional shortening (FS) observed 1 week post-MI in MI and MI/AAVGFP, which progressively decreased at 4 and 8 weeks (Figure 2B). Decreased FS was associated with increased left ventricular end-systolic and end-diastolic dimension (LVESD and LVEDD) (Figures 2C and 2D). AAVAng-(1-9) infusion significantly attenuated reduced FS at all time points. At 8 weeks, FS in MI/AAVAng-(1-9) was significantly reduced compared to sham (38.5 ± 1.9% vs. 49.1 ± 1.6%; p < 0.05), although it was significantly increased compared to MI and MI/AAVGFP (MI = 25.8 ± 2.2%; MI/AAVGFP = 26.6 ± 0.7%; p < 0.05). Importantly, in MI/AAVAng-(1-9), FS remained stable from 1 week, in contrast to the progressive decline in other groups (Figure 2B). At 1 week, LVESD in MI/AAVAng-(1-9) was significantly reduced compared to MI/AAVGFP (Figure 2C). No significant changes in posterior left ventricular (LV) wall thickness were detected at any time point (Figure 2E). Additionally, ejection fraction (EF) was significantly reduced 1 week post-MI in MI and MI/AAVGFP and further decreased at 4 and 8 weeks (Figure 2F). AAVAng-(1-9) delivery significantly attenuated reduced EF at all time points. E/A wave ratio was not different between groups (Figure 2G).

**Figure 3:**
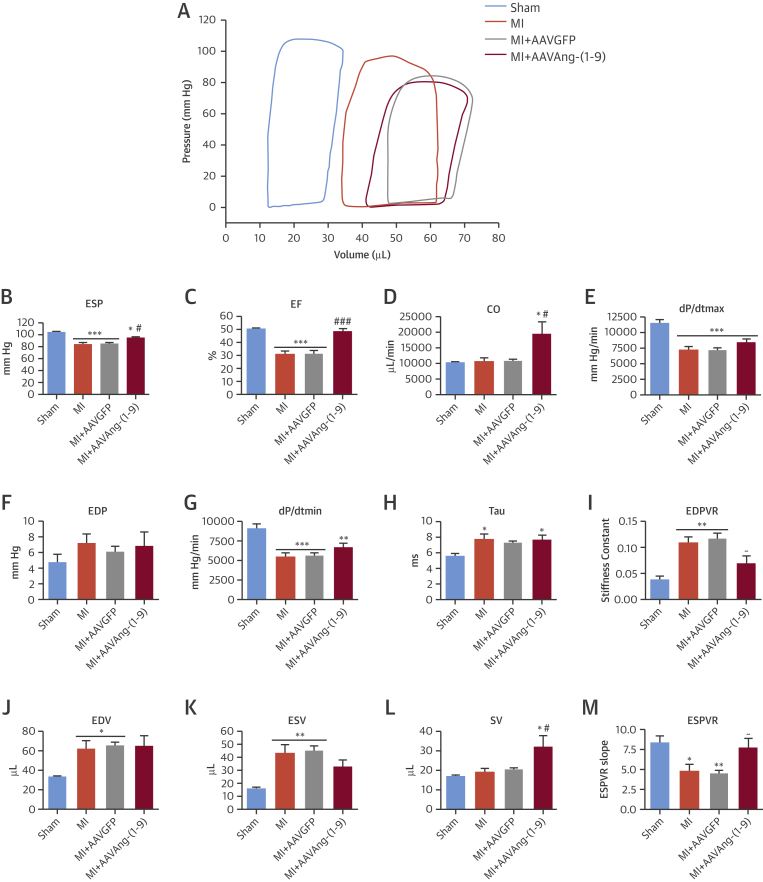
Hemodynamic Indexes LV hemodynamic measurements at 8 weeks were determined using a PV-loop system with true blood volume calculated using Wei’s equation. Shown are **(A)** PV-loop relationship example; the systolic functional indexes of **(B)** ESP, **(C)** EF, **(D)** CO; and **(E)** dP/dt_max_, the diastolic functional indexes of **(F)** end-diastolic pressure (EDP), **(G)** dP/dt_min_, **(H)** Tau, and **(I)** EDPVR; and the volume indexes of **(J)** EDV, **(K)** ESV, **(L)** SV, and **(M)** ESPVR. *p <0.05, **p <0.01, ***p <0.001 versus sham; ^#^p <0.05, ^##^p <0.01, ^###^p <0.001 versus MI and MI/AAVGFP; ^∼^p <0.05 versus MI/AAVGFP only. n = 9, 9, 9, and 8 for, sham, MI, MI/AAVGFP, and MI/AAVAng-(1-9), respectively. Data presented as mean ± SEM. CO = cardiac output; EDP = end diastolic pressure; EDPVR = EDP volume relationship; EDV = end diastolic volume; ESP = end systolic pressure; ESPVR = ESP volume relationship; ESV = end systolic volume; PV = pressure volume; SV = stroke volume; other abbreviations as in Figures 1 and 2.

Eight-week PV loop measurements in MI/AAVAng-(1-9) revealed significant attenuation of the decreased systolic indexes observed in MI and MI/AAVGFP (Figure 3A). AAVAng-(1-9) significantly increased end-systolic pressure (Figure 3B) (p < 0.001), EF (Figure 3C) (p < 0.001), and cardiac output (CO) (Figure 3D) (p < 0.05). Importantly, EF was normalized to sham level, whereas CO was significantly increased compared to sham (p < 0.05). However, maximum derivative of change in systolic pressure over time (dP/dt_max_) remained significantly reduced to 78.5% of sham (Figure 3E) (p < 0.001). There were no significant differences in end-diastolic pressure, dP/dt_min_, and the rate constant of LV pressure decline (Tau) following AAVAng-(1-9) delivery (Figures 3F to 3H). The end-diastolic pressure volume relationship (EDPVR) in MI and MI/AAVGFP was significantly increased (p < 0.05) to 363.3% and 400% of sham, respectively (Figure 3I). Following AAVAng-(1-9), EDPVR was normalized to sham levels (Figure 3I), while there was no detectable change in end-diastolic volume (Figure 3J). End-systolic volume was significantly increased in MI and MI/AAVGFP (p < 0.01); however, it was not different between sham and MI/AAVAng-(1-9) (Figure 3K). Stroke volume was significantly increased (p < 0.05) in MI/AAVAng-(1-9) compared to sham and MI/AAVGFP (Figure 3L). Additionally, the end-systolic pressure volume relationship (ESPVR) was significantly decreased in MI and MI/AAVGFP but normalized by AAVAng-(1-9) (Figure 3M).

### Effects on hypertrophy and fibrosis

**Figure 4:**
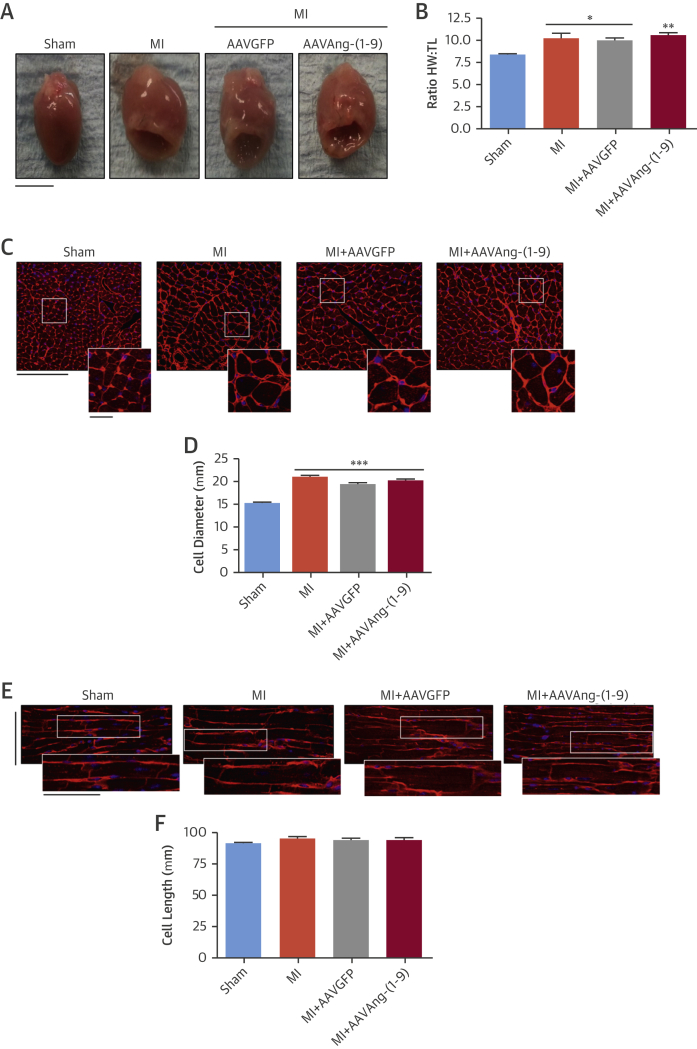
Cardiomyocyte Hypertrophy **(A)** Heart images at 8 weeks (scale bar = 5 mm). **(B)** Ratio of HW to TL. *p <0.05, **p <0.01 versus sham. n = 10, 10, 9, and 8 for, sham, MI, MI/AAVGFP, and MI/AAVAng-(1-9), respectively. Data presented as mean ± SEM. **(C)** Cardiac cross sections in transverse axis (original main image magnification ×25; scale = 50 μm; inset zoom image scale = 12.5 μm). **(D)** LV cardiomyocyte diameter in hearts. ***p <0.001 versus sham. n = 10, 10, 9, and 8 for sham, MI, MI/AAVGFP, and MI/AAVAng-(1-9), respectively. **(E)** Cardiac cross sections in longitudinal axis (original main image magnification = ×25; scale = 50 μm; inset zoom image scale = 50 μm). **(F)** LV cardiomyocyte length. n = 10, 10, 9, and 8 for sham, MI, MI/AAVGFP, and MI/AAVAng-(1-9), respectively. Data presented as mean ± SEM with average cell size taken as average of a group of cells evenly distributed across LV. HW = heart weight; TL = tibia length; other abbreviations as in Figures 1 and 2.

**Figure 5:**
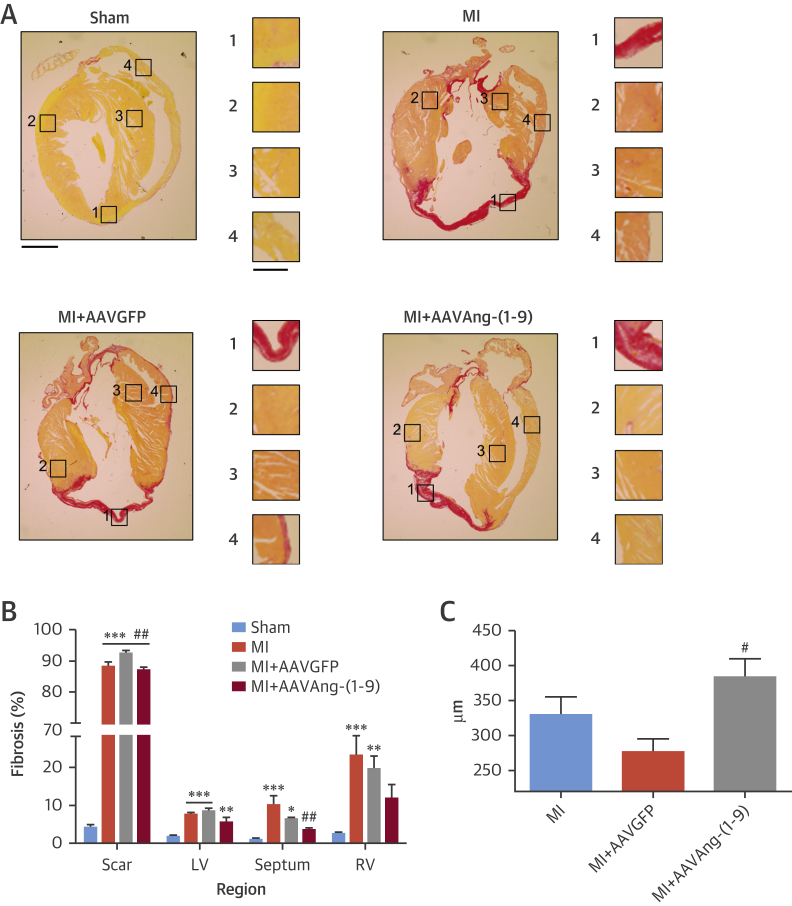
Cardiac Fibrosis **(A)** Picrosirius red staining of heart sections (original magnification ×1.25; scale = 1 mm; zoom insert image scale = 0.5 mm). **(B)** Quantification of total cardiac fibrosis of the scar, LV, right ventricular, and septum regions. **(C)** Scar thickness for each MI group. n = 10, 10, 9, and 8 for sham, MI, MI/AAVGFP and MI/AAVAng-(1-9), respectively. Data presented as mean ± SEM. *p <0.05, **p <0.01, ***p <0.001 versus sham region; ^#^p <0.05, ^##^p <0.01 versus MI and MI/AAVGFP region. Abbreviations as in Figures 1 and 2.

Heart weight/tibia length (HW:TL) ratios were significantly increased in all MI groups to 121%, 118%, and 125% of sham for MI, MI/AAVGFP (p < 0.05), and MI/AAVAng-(1-9) (p < 0.01), respectively (Figures 4A and B). Cardiomyocyte diameter was significantly increased compared to sham in all MI groups (sham: 15.1 ± 0.3 μm; MI: 20.9 ± 0.5 μm; MI/AAVGFP: 19.4 ± 0.4 μm; MI/AAVAng-(1-9) = 20.2 ± 0.4 μm; p <0.001) (Figures 4C and D). No significant differences in cell length were observed (Figures 4E and F). LV and right ventricular fibrosis was significantly increased in all MI groups (p < 0.01) (Figures 5A and B). Septal fibrosis in MI and MI/AAVGFP was significantly increased compared to sham, but significantly reduced in MI/AAVAng-(1-9) (MI: 10 ± 2.4; MI/AAVGFP: 6.3 ± 0.4; MI/AAVAng-(1-9): 3.4 ± 0.6%; p < 0.01). Perivascular fibrosis was significantly elevated in MI and MI/AAVGFP; however, delivery of AAVAng-(1-9) normalized this (Online Figure 1). Scar size was consistent among all MI groups (MI: 35.9 ± 2.8%; MI/AAVGFP: 35.2 ± 2.1%; MI/AAVAng-(1-9): 36.9 ± 2.5%) (data not shown). However, in MI and MI/AAVGFP, scar thickness was 329 ± 25 μm and 276 ± 3.9 μm, respectively, whereas in MI/AAVAng-(1-9), scar thickness was significantly increased versus MI/AAVGFP to 383 ± 14 μm (p < 0.05) (Figure 5C).

Quantitative polymerase chain reaction of levels of RAAS genes in cardiac complementary DNA revealed significantly increased ACE in all MI groups compared to sham, whereas ACE2 expression remained unchanged (Online Figures 2A and 2B). Furthermore, significantly increased AT_2_R expression in MI/AAVAng-(1-9) was observed, while the angiotensin type 1 receptors were significantly decreased in all MI groups (Online Figures 2C and 2D). Mas expression was significantly downregulated in MI/AAVAng-(1-9) (Online Figure 2E). There were no significant changes in gene expression of the inflammatory markers tumor necrosis factor alpha; interleukin (IL) 1β, IL6, or IL12a; or interferon γ (Online Figure 3). Additionally, gene expression of matrix metalloproteinase (MMP)-2 and -12 and tissue inhibitor of metalloproteinase-1 were significantly increased in MI groups compared to sham, whereas MMP-9 and -14 were not changed (Online Figure 4). MMP-2 was significantly reduced in MI/AAVGFP and MI/AAVAng-(1-9) and MMP-12 was significantly reduced in MI/AAVAng-(1-9). SERCA2a was significantly reduced in all MI groups (Online Figure 5).

### Effects in cardiomyocytes and whole hearts

**Figure 6:**
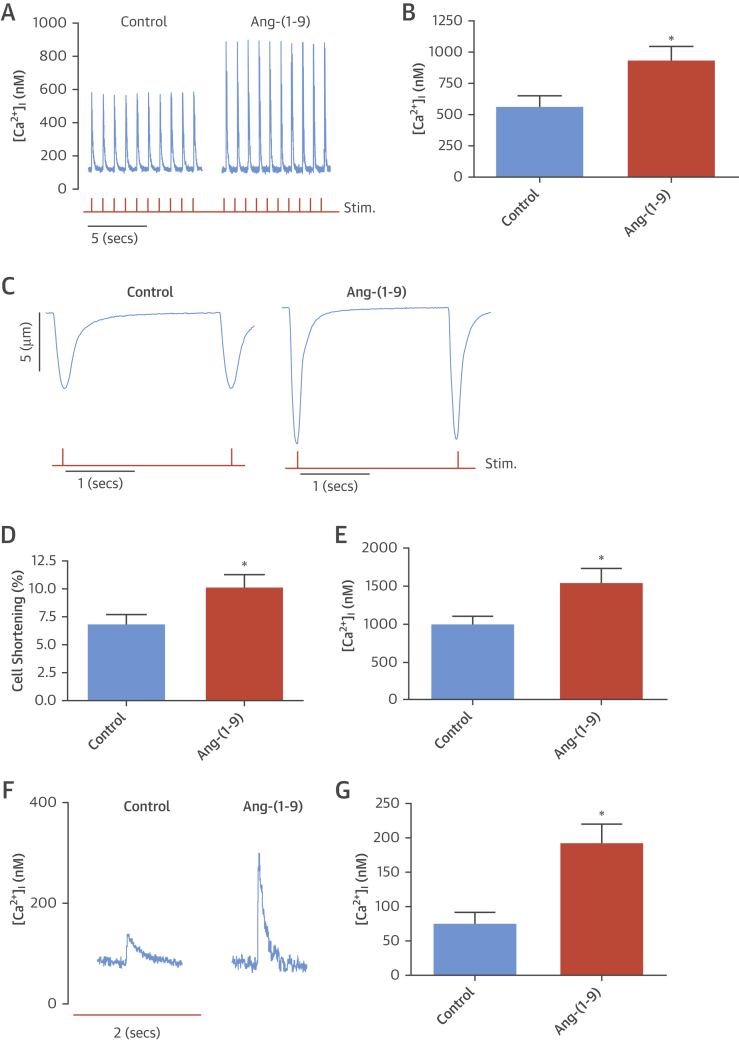
Excitation Contraction Coupling in Isolated Cardiomyocytes **(A)** Representative Ca^2+^-transient traces from Ang-(1-9)–treated cells. **(B)** Average Ca^2+^-transient amplitude for cardiomyocytes untreated (control; n = 21) or pre-treated with 1 μmol/l Ang-(1-9) (n = 21). **(C)** Cell shortening traces. **(D)** Cell shortening. **(E)** Average sarcoplasmic reticulum Ca^2+^ content. *p < 0.05 versus control. **(F)** Average first Ca^2+^ transient traces after 10 mmol/l caffeine. **(G)** Average L-type Ca^2+^-transient amplitude [control, n = 10; Ang-(1-9), n = 11]. *p < 0.05 versus control. Data presented as mean ± SEM. Red trace = 1 Hz stimulation. Abbreviations as in Figure 1.

Calcium (Ca^2+^) handling, in particular sarcoplasmic reticulum (SR)–mediated Ca^2+^ release, is the major determinant of cardiomyocyte contractility. Therefore, characteristics of SR-mediated Ca^2+^ release and uptake (Ca^2+^ transients) were determined in murine cardiomyocytes acutely exposed to soluble Ang-(1-9) peptide. Ang-(1-9) significantly increased Ca^2+^ transient amplitude (control: 561.0 ± 86.5 nmol/l; Ang-(1-9): 933.7 ± 107.0 nmol/l; p <0.05) (Figures 6A and 6B); an observation also observed in cardiomyocytes isolated from MI hearts (Online Figure 6). In parallel, Ang-(1-9) significantly increased cell shortening compared to control cardiomyocytes [control: 6.8 ± 0.9%; Ang-(1-9): 10.2 ± 1.1%; p < 0.05] (Figures 6C and 6D). The rate of decline of the Ca^2+^ transient was not significantly altered by Ang-(1-9) (data not shown), suggesting no change in rate of Ca^2+^ removal from the cytosol through SR uptake via SERCA or the sodium calcium exchanger. To determine SR Ca^2+^ content, a major determinant of Ca^2+^ transient amplitude (20), a rapid bolus of 10 mmol/l caffeine was applied at the end of the protocol to release all SR Ca^2+^ into the cytosol. The caffeine-induced Ca^2+^-transient amplitude in Ang-(1-9)–incubated cardiomyocytes was significantly increased compared to control, indicating an increased SR Ca^2+^ content (control: 987.5 ± 101.4 nmol/l; Ang-(1-9): 1,535.2 ± 188.8 nmol/l; p < 0.05) (Figure 6E). SERCA-mediated Ca^2+^ uptake is bypassed during application of 10 mmol/l caffeine and cytosolic Ca^2+^ removal occurs predominately via the sodium calcium exchanger. The rate constant of decline of the caffeine-induced Ca^2+^-transient (Tau) was unaltered by Ang-(1-9), supporting the conclusion that Ang-(1-9) does not alter cardiomyocyte Ca^2+^ extrusion (Online Figure 7). One possible route through which the SR Ca^2+^ content and transient could be elevated is through increased influx of Ca^2+^ (e.g., via L-type Ca^2+^ channels). To assess this, cardiomyocytes were continuously perfused with Ang-(1-9) and a 10 mmol/l bolus of caffeine applied for 10 s after 15 min followed by 2 min of steady state measurements while cells were stimulated. The amplitude of the first Ca^2+^ transient after caffeine was taken as an index of Ca^2+^ influx via the L-type Ca^2+^ channel 21, 22, 23. Ang-(1-9) significantly increased the L-type Ca^2+^-transient amplitude versus controls (191.8 ± 28.4 nmol/l vs. 74.6 ± 17.3 nmol/l; p < 0.05) (Figures 6F and 6G).

**Figure 7:**
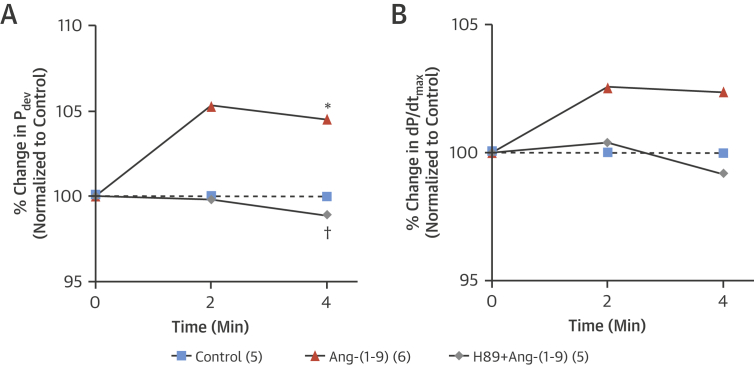
Inotropic Effects of Ang-(1-9) Upon achieving maximum developed pressure, hearts were paced at 320 beats/min and allowed to reach steady state for 10 min before addition of 1 μmol/l Ang-(1-9). The protein kinase A inhibitor H89 (1 μmol/l) was perfused 10 min prior to adding Ang-(1-9) and was present throughout perfusion. **(A)** There were significant differences in **(A)** LV developed pressure compared to control hearts, but not in **(B)** the first derivative of LV developed pressured (dP/dt_max_). Control n = 5, Ang-(1-9) n = 6, and H89 + Ang-(1-9) n = 5. *p <0.05 versus control hearts; †p <0.05 versus Ang-(1-9). Abbreviations as in Figure 1.

To assess whether the positive inotropy observed in isolated cardiomyocytes translated to whole heart contractile function, hearts isolated according to the Langendorff model were perfused with Ang-(1-9). After 4 min of perfusion, Ang-(1-9) induced a significant increase in developed pressure with a concomitant elevation in dP/dt_max_, confirming a positive inotropic response to Ang-(1-9) (Figure 7). Since protein kinase A (PKA) has been previously reported to modulate calcium flux via the L type Ca^2+^ channel following application of Ang-(1-7) (24), we used the inhibitor H-89, which did indeed abolish the response to Ang-(1-9), thus supporting a role for PKA in the positive inotropic effect of Ang-(1-9).

**Figure 8:**
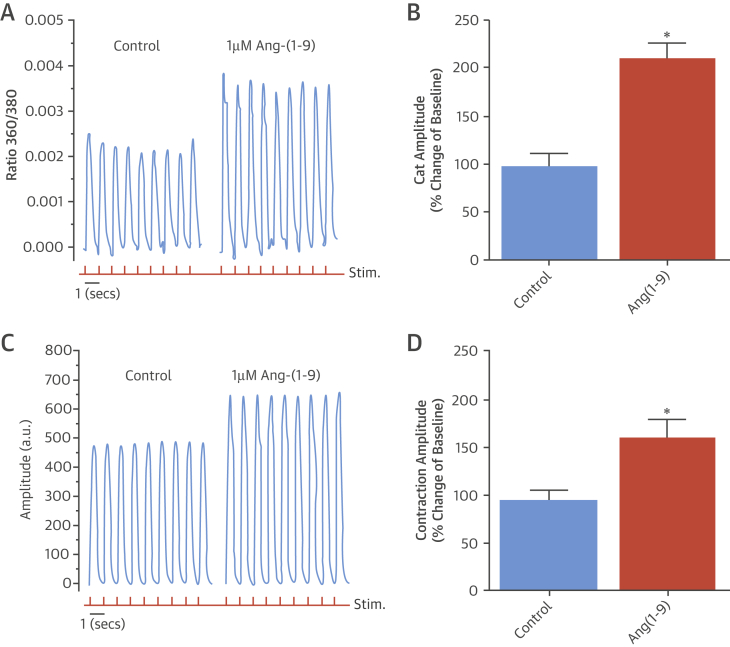
Excitation Contraction Coupling in hiPSC-CMs **(A)** Representative Ca^2+^-transient traces from Ang-(1-9)-treated cells. **(B)** Average Ca^2+^-transient amplitude for iCell^2^ hiPSC-CM (Cellular Dynamics International, Madison, Wisconsin). **(C)** Contractility traces. **(D)** Average contraction amplitude for iCell^2^ hiPSC-CMs. *p <0.05 versus control. Data presented as mean ± SEM (n = 5). Red trace = 1 Hz stimulation. hiPSC-CM = human-induced pluripotent stem cell-derived cardiomyocytes; other abbreviation as in Figure 1.

To extrapolate the findings in murine cardiomyocytes and rat hearts to a human model, hiPSC-CMs were used (25), and intracellular Ca^2+^ and contraction were measured before (baseline) and after 15 min incubation with different Ang-(1-9) concentrations. A dose-dependent increase in Ca^2+^ transient and contraction amplitudes was observed within concentrations 0.5 μmol/l to 2 μmol/l (data not shown) with no effect on parameters such as calcium transient upstroke, rate of decline, or contraction/relaxation times. Ca^2+^ transient amplitude and contraction following incubation with 1 μmol/l Ang-(1-9) compared to control cells was measured and a 210 ± 10% change from baseline in Ca^2+^ transient amplitude for cells incubated with 1 μmol/l Ang-(1-9) was observed, an effect significantly different from control cells (98 ± 13% change from baseline) (Figures 8A and B). A parallel effect was observed for contraction in terms of increased amplitude (160 ± 13% vs. 93 ± 12%) (Figures 8C and D).

## Discussion

Our study focused on an innovative gene therapy approach to deliver Ang-(1-9) directly to the heart to assess therapeutic effects and mechanisms of action in a murine MI model. AAV9-mediated delivery of Ang-(1-9) reduced acute rupture and mildly affected cardiac hypertrophy and fibrosis, but preserved LV systolic function, even at 8 weeks post-MI (Central Illustration). The effects of Ang-(1-9) were mediated via a direct positive inotropic effect. In isolated cardiomyocytes, Ang-(1-9) enhanced Ca^2+^ handling by increasing SR Ca^2+^ content and Ca^2+^ transient amplitude (Central Illustration).

While rupture rates in MI and MI/AAVGFP groups were consistent with previous studies 26, 27, AAVAng-(1-9) reduced acute rupture. Although the reasons for this are not entirely clear, because Ang-(1-9) delivery increased scar thickness, the mechanism underlying this effect might entail stabilization of cardiac architecture in the acute phase post-MI. This is a potentially beneficial finding because overall incidence of cardiac rupture in acute ST-elevation MI patients is 6.4% (28). At 8 weeks, there were no detectable differences in gene expression of tumor necrosis factor alpha, IL1β, IL6, IL12α, and interferon-γ associated with inflammation; this is not unexpected because these cytokines are upregulated acutely following MI (29). We also measured expression of genes involved in tissue remodeling in MI, including MMP-2, -9, -12, -14 and tissue inhibitor of metalloproteinase-1 (30). Differences in MMP-2 and -12 could be detected at 8 weeks following AAVAng-(1-9) delivery, suggesting one possible mechanism by which Ang-(1-9) can modulate remodeling during scar evolution. Understanding how AAVAng-(1-9) delivery contributes to healing post-MI and scar thickening will be important to investigate by assessing a range of acute time points within the first few days post-delivery, when inflammation is high and the scar is rapidly remodeling and evolving. Since AAV-mediated delivery has been detectable as early as 2 days post-delivery and is accelerated in damaged tissue 31, 32, 33, future studies may reveal other mechanisms of AAVAng-(1-9) action.

AAVAng-(1-9) delivery significantly reduced fibrosis, although not specifically in the LV, suggesting this did not significantly contribute to Ang-(1-9)’s inotropic effect. The reduced fibrosis (albeit regional) aligned with previous studies where osmotic mini-pump delivery attenuated cardiac fibrosis (8). This supports a general antifibrotic effect for the counter-regulatory RAAS axis, given the ACE2/Ang-(1-7)/Mas system’s well-established antifibrotic effects on the myocardium 34, 35. Ang-(1-9) did not mediate any antihypertrophic effect, contrary to previous reports 9, 11, possibly because the previous work only assessed hypertrophy at 2 weeks compared to at 8 weeks here. Therefore, early acute effects of Ang-(1-9) on limiting hypertrophy might not be maintained once significant structural remodeling has taken place at 8 weeks.

AAVAng-(1-9)–transduced hearts consistently had greater contraction and blood ejection, evidenced by dramatically increased CO and stroke volume and normalized EF, showing that regardless of MI-induced dilation, function was maintained. This contrasted with a previous study assessing osmotic mini-pump-mediated Ang-(1-9) delivery on cardiac function in rats post-MI that showed significantly reduced LV dimensions and volumes and reported reduced wall thickness, but no change in LV systolic function (9). Therefore, while certain parameters were consistent (e.g., alterations in LVESD), this current study clearly demonstrated markedly improved systolic function with AAVAng-(1-9), corroborated via echocardiography and PV loop measurements. A major reason for the difference may be method of peptide delivery: Direct gene transfer in the heart via AAV9 utilized here (vs. osmotic mini-pump) achieved high local cardiac concentrations 17, 36. Local tissue-specific effects of the RAAS might differ from systemic effects; for instance, local Ang II production in the heart does not produce acute cardiac remodeling, whereas systemic infusion does (37). Tissue-specific effects were also reported for Ang-(1-7) in MI in transgenic mice (38), and lentiviral delivery of Ang-(1-7) in rat MI improved cardiac function (39), supporting the concept that local cardiac Ang-(1-7) and Ang-(1-9) produce beneficial effects.

Additionally, AAVAng-(1-9) delivery significantly increased myocardial AT_2_R gene expression. AT_2_R expression is reported to increase acutely following MI (40). Given that AT_2_R is associated with cardioprotective effects (41), including reduced remodeling and improved function post-MI, this might underlie some therapeutic effects of AAVAng-(1-9). A small but significant change in Mas expression was also observed in the MI/AAVAng-(1-9) group. The reason for this is not clear because Mas is upregulated in dysfunctional hearts 4 weeks post-MI in rats (42); however, since in our studies, cardiac function was preserved in AAVAng-(1-9)-infused mice, Mas downregulation might be compensatory. This requires confirmation.

To gain further insight into potential mechanisms underlying the positive inotropic effects of Ang-(1-9), excitation contraction coupling was studied in isolated murine cardiomyocytes (normal and after MI) and the whole rat heart and hiPSC-CMs. We demonstrated a direct inotropic effect of Ang-(1-9), mediated through increasing Ca^2+^ transient amplitude leading to increased contraction, and possibly explained via increased L-type Ca^2+^ influx paralleled by increased SR Ca^2+^ content. Although a direct inotropic effect of Ang-(1-9) has not been reported previously, when Ang-(1-7) is applied intracellularly to cardiomyocytes, PKA is activated, leading to increased L-type Ca^2+^ channel activity (24). Ang II is reported to increase Ca^2+^ transient amplitude and intracellular Ang II is reported to increase Ca^2+^ transient amplitude via modulating L-type Ca^2+^ current and releasing SR Ca^2+^ 43, 44. Several cardiomyocyte Ca^2+^ handling proteins, including the L-type Ca^2+^ channel, are regulated by PKA-mediated pathways (45). The increased contractility in the isolated hearts perfused with Ang-(1-9) in this study and the effect of PKA inhibition suggest that Ang-(1-7) and Ang-(1-9) may act by similar mechanisms leading to PKA activation.

### Study limitations

Our studies were performed in a murine model of permanent LAD ligation and future studies in larger animal models following ischemic reperfusion would be helpful to inform translation of the gene therapy. Furthermore, the inotropic effects studied in isolated cardiomyocytes were performed via peptide perfusion and further work to isolate cardiomyocytes from hearts infused in vivo with the gene therapy combined with use of patch clamping would enable full dissection of the inotropic effects of Ang-(1-9). Nonetheless, the current studies strongly support a beneficial effect of cardiac Ang-(1-9) gene therapy in the setting of MI.

## Conclusions

This study suggested that gene therapy to augment Ang-(1-9) levels in the heart produces clear benefit in a murine MI model. Our data supported the notion that administration of the counter-regulatory RAAS peptide Ang-(1-9) via translational gene therapy is a novel and promising approach in heart disease that preserves cardiac systolic function post-MI and is maintained in a sustained manner.

Perspectives **COMPETENCY IN MEDICAL KNOWLEDGE:** In a preclinical model of MI, gene therapy with Ang-(1-9) preserved systolic function by mediating a direct positive inotropic effect on cardiomyocytes.

**TRANSLATIONAL OUTLOOK:** Further work is needed to assess whether Ang-(1-9) gene delivery in other large animal models of myocardial infarction preserves systolic function and prevents heart failure.

## Figures and Tables

**Central Illustration:**
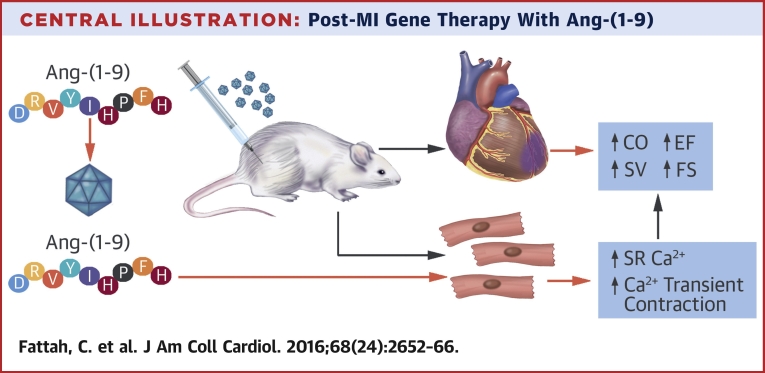
Post-MI Gene Therapy With Ang-(1-9) Adeno-associated virus serotype 9–mediated delivery of Ang-(1-9) via tail vein in a murine model of MI following coronary artery ligation produced significantly improved CO, SV, EF, and FS. Incubating freshly isolated adult murine cardiomyocytes or human induced pluripotent stem cell-derived cardiomyocytes with Ang-(1-9) leads to elevated SR Ca^2+^ content through stimulation of the L type calcium channel and enhanced contraction. Ang-(1-9) = angiotensin-(1-9); CO = cardiac output; EF = ejection fraction; FS = fractional shortening; MI = myocardial infarction; SR = sarcoplasmic reticulum; SV = stroke volume.
