# Supplementary material for: Gene Therapy With Angiotensin-(1-9) Preserves Left Ventricular Systolic Function After Myocardial Infarction
Source: J Am Coll Cardiol. 2016 Dec 20;68(24):2652–66. doi: 10.1016/j.jacc.2016.09.946 (PMC5158000; doi:10.1016/j.jacc.2016.09.946)
Supplement: Online Data [file mmc1.docx]

**Methods**

**Production of viral vectors**

We previously reported generation of a fusion protein expression cassette for production of Ang-(1-9) from adenoviral vectors (1). Here, the expression cassette was sub-cloned into pAAV-MCS [pAAV-MCS-Ang-(1-9)] and plasmid DNA purified using Qiagen Plasmid Maxi Kit (Qiagen, UK) following the manufacturer’s guidelines for production of AAVAng-(1-9) vectors. Briefly, HEK293-T cells were co-transfected with pAAV-MCS-Ang-(1-9), the p5E18-VD2/9 plasmid containing genes for AAV2 Rep and AAV9 capsid proteins (2) and the pHELPER plasmid expressing the three Adenovirus helper genes (Stratagene). AAV9 vectors were released from cells 48 hr post-transfection *via* freeze-thaw prior to being purified using PEG precipitation and CsCl density gradient centrifugation (3,4). Titers were determined using viral genome copy number *via* pooled, dialyzed gradient fractions by real-time PCR against a standard curve of a plasmid containing the vector genome (3). Titers ranged from 2.5x10^12^ to 7.4x10^13^ viral genome copies per mL (vg/mL). AAVGFP control vectors were prepared in an identical fashion, exchanging AAV-MCS-Ang-(1-9) for AAV-MCS-eGFP in the co-transfection.

**Animal experiments**

All surgical procedures were performed in accordance with the Animals Scientific Procedures Act (1986) and were approved by the University of Glasgow Animal Welfare and Ethical Review Panel and the UK Home Office. Male C57BL/6 mice (Envigo, UK) were housed under controlled environmental conditions (12 hr light/ dark cycles at ambient temperature and humidity) and maintained on a standard chow diet.

**Mouse model of myocardial infarction (MI) and delivery of AAV vectors**

MI procedures were performed in 9-11 wk old mice (body weight ~25 g) under aseptic technique. Mice received intra-peritoneal injections of 0.6 μg Buprenorphrine and 0.1 mg Carprofen in 0.4 mL of sterile 0.9 % NaCl saline. Animals were anaesthetized with 4% isofluorane, a diagonal incision made in the skin over the rib cage, blunt dissection of muscles performed and a thoracotomy performed at the 5^th^ intercostal rib space. For animals undergoing MI, the LAD was ligated using 9-0 ethilon suture (Ethicon, Livingston, UK), visualized by immediate blanching of the myocardium. Sham animals had identical procedures, without ligation of the LAD. Animals were recovered, monitored daily and received 25 µg of Temegesic (Buprenorphrine) in soft food for 72 hr following surgery. AAVAng-(1-9) or AAVGFP were delivered intravenously via tail vein injection following MI. A total of 1x10^11^ VG in a total volume of 100 μL per animal was delivered in similarity to previous studies (5).

**Assessment of cardiac function *via* echocardiography or pressure volume loop**

Echocardiography was performed weekly (**Figure 1A**) using a Siemens Acuson Sequoia 512 ultrasound unit with an 18LS probe set at a frequency of 14 MHz. Animals were anesthetised in 4% isofluorane mixed with oxygen. They were placed on a nose cone on a heat mat and isofluorane was reduced to 1.5% at a flow rate of 1 L/min for the duration of the measurements. Pressure volume (PV) loop measurements were made using an ADVantage AD500 PV-loop system (Transonic Systems Inc, The Netherlands). Animals were anaesthetised, a vertical incision made in the neck and the right carotid artery blunt dissected A PV catheter was inserted into the right carotid artery and advanced into the left ventricle (LV) which was confirmed by a drop in end diastolic pressure (EDP). Once steady state was reached, a vena cava occlusion was performed to assess LV stiffness and diastolic dysfunction.

**Histological analysis**

Tissues were paraffin embedded and 4 μm sections cut. Fibrosis was assessed by picrosirius red staining as previously described (6). Wheat germ agglutinin (WGA) was performed using WGA-AlexaFlour555 (Life Technologies, Paisley). Briefly sections were blocked for 1 hr in 1% BSA and 5% goat serum and then incubated for 1 hour at room temperature in 10 μg/mL WGA. EGFP expression was visualized on 4 μm formalin fixed sections by immunohistochemistry with a 1:200 dilution of anti-rabbit-eGFP antibody (Vector Biolabs; clone BA-1000) and a goat-anti-rabbit secondary antibody conjugated to horse radish peroxidase at 1:200 dilution and detected using Vectastain ABC reagent (Vector Laboratories, Peterborough, UK). Alternatively, EGFP levels were quantified in cardiac lysates using a Wallac Victor 2. For quantification of histology either an EVOS FL colour imaging system (Life Technologies, Paisley, UK) or a Zeiss laser scanning confocal microscope (LSM 510 Meta, Carl Zeiss Ltd., Cambridge, UK) using Diode 405-30 (excitation of DAPI) and HeNe 543 (excitation of WGA) lasers were used. WGA analysis was only performed on the LV and an arbitrary minimum cell length cut off of 70 µm was applied to account for not being able to observe the whole cell given the optical sectioning of the confocal microscope. Staining quantification was performed using ImageJ. For whole heart sections, quantification was performed in each heart region (scar, LV, RV and septum) with an equivalent apical section as control in sham animals. Images were split into a red-green-blue (RGB) image stack. For picrosirius red staining, quantification was performed on the green image. Colour-intensity threshold included all positive-stained pixels and was quantified as the % of total pixels within the intensity threshold of a selected area of interest. For vessel-associated staining quantification, images were analysed in the same way with the region of interest limited to any vessels visible in the image.

**Quantitative RT-PCR**

Tissue was snap frozen in Qiazol and RNA was extracted from tissues using an RNeasy mini kit (Qiagen, UK) following the manufacturer’s instructions and reverse transcribed to cDNA using SuperScipt (Invitrogen, Paisley, UK): (per reaction) 500 ng RNA, 1 x reaction buffer, 2 mM MgCl_2_, 2.5 mM each dNTP, 50 µM random hexamers, 18 U RNAse inhibitor and 50 U reverse transcriptase. Taqman inventoried gene expression assays [Agtr1a (AT_1_R), Mm01957722_s1; Agtr2 (AT_2_R), Mm01341373_m1; Mas1, Mm00434823_s1; ACE, Mm00802048_m1; ACE2, Mm01159003_m1; ATP2A2 (SERCA2a), Mm01201431_m1; MMP-2, Mm00439493_m1; MMP-14, Mm00485054_m1; MMP-12, Mm00500554_m1; MMP-9, Mm00442991_m1; TNFα, Mm00443258_m1; IL-12α, Mm00434165_m1; IFNg, Mm01168134_m1; TIMP-1, Mm00441818_m1; IL-6, Mm00446190_m1; IL-1β, Mm00434228_m1; GAPDH, 4352339E] were purchased from Applied Biosystems (UK) and analysed on a 7900HT Fast Real-Time PCR System (Invitrogen, Paisley, UK).

**Isolated cardiomyocyte calcium and shortening measurements**

Mouse ventricular cardiomyocytes were isolated from the hearts of adult C57BL/6 mice *via* constant-flow Langendorff retrograde perfusion with collagenase I (Worthington Chemicals, USA) and protease type XIV (Sigma-Aldrich, Poole, UK) as per previous publications (7). Calcium imaging was performed on an inverted epifluorescence microscope using a spinning wheel monochromator (Cairn Research, Faversham, UK) switching between excitation wavelengths of 340 and 380 nm at a rate of 250 Hz. All experiments were performed at 37°C. Cell stimulation was at 1.0 Hz and cells were loaded with Fura-4F AM ester ratiometric dye (Life Technologies, Paisley, UK). Experiments were carried out at 1.8 mM [Ca^2+^] in a HEPES buffered superfusate (10 x HEPES buffered perfusate (in mmol/L): NaCl 140.0, KCl 4.0, MgCl_2_ 1.0, HEPES 5.0, glucose 11.1, CaCl_2_ 1.8. pH 7.4 at 37°C with NaOH). Sarcoplasmic reticulum (SR) calcium content was determined using a rapid application of 10 mM caffeine. For Ang-(1-9) pre-treatment, Fura-4F loaded cells were either pre-incubated for 15 min at room temperature with 1 µM Ang-(1-9) (Phoenix Pharmaceuticals) and perfused with HEPES superfusate containing 1 µM Ang-(1-9) throughout or stimulated for 2 min at 1 Hz for steady state recordings (control) before stimulation of cells with 1 μM Ang-(1-9). Steady-state recordings were collected for 3 min before cessation of stimulation and the 10 mM caffeine bolus. For Ca^2+^ imaging, fluorescence was sampled at 5 KHz and data recorded in Clampex 10.3 software using an analogue to digital convertor (Axon Instruments, California, USA). Data was imported into Origin 6.1 software (Originlab, Massachusetts, USA) and 340/380 ratio determined. A steady-state recording of 12 transients were used to calculate average calcium transient parameters. Fluorescence ratio was converted into intracellular calcium concentration ([Ca^2+^]_i_) as previously described (8). Cell shortening was measured simultaneously using edge-detection imaging equipment (IonOptix, Dublin, Ireland) and accompanying software (Ionwizard 6.0) which measured the change in cell length during stimulation. Data was sampled at 2.0 KHz and analysed using Origin 6.1 software. An equivalent length of trace to Ca^2+^ transient analysis was averaged (12 contractions). Diastolic cell length (L_0_) was taken immediately before stimulation and used to calculate mean fractional shortening.

**Isolated whole heart LV pressure measurements**

Male adult Wistar rats weighing ~250 g were sacrificed by cervical dislocation. Hearts were quickly excised and Langendorff perfused (9) at constant temperature (37°C) and constant flow (10 mL/min)(9) with Tyrode’s solution (mM: 116.0 NaCl, 20.0 NaHCO_3_, 0.4 Na_2_HPO_4_, 1.0 MgSO_4_-7H_2_O, 5.0 KCl, 11.0 glucose, 1.8 CaCl_2_) bubbled with 95% O_2_, 5% CO_2_ (pH 7.4). A fluid-filled cling film balloon was inserted into the LV and connected to a solid state pressure transducer (Scisense, London, Canada). The balloon was inflated to give an end-diastolic pressure (P_min_) of ~3-5 mmHg. Hearts were paced at 320 bpm and allowed to reach a steady state for 10 min before the addition of 1 μM Ang-(1-9) for a further 4 min. The PKA inhibitor H89 (1 μM) was perfused 10 min prior to the addition of Ang-(1-9) and then was present throughout the period of perfusion of Ang-(1-9) as previously described (8). LV peak pressure (P_max_), LV developed pressure (P_dev_), the first derivative of LV developed pressure (d*P*/dt_max/min_) and the exponential decay of LV pressure (*tau*), were measured using LabChart 7.0 (ADInstruments, Oxford, UK).

**Human induced pluripotent stem cell derived cardiomyocytes (hiPSC-CMs) culture**

hiPS-CMs (iCell^2^™ Cardiomyocytes), cell culture thawing and maintenance media were purchased from Cellular Dynamics International (cat# CMC-100-012-001) (Madison, WI). The cells were cultured in 96-well glass bottom plates according to manufacturer instructions (http://www.cellulardynamics.com). Briefly, iCell^2^ cardiomyocytes (hiPS-CMs) were supplied cryopreserved at a density of ~5.0 x 10^6^/ml and stored at –150°C until use. Cryopreserved cells were thawed in a 37^o^C water bath without shaking for 3 minutes, transferred to a 50 mL falcon tube in a drop wise manner, before addition of 9 mL iCell thawing media. Viability was determined using a manual haemocytometer. Cells were seeded at 1.6x10^5^cells/cm^2^ in thawing media and incubated at 37^o^C with 5% CO_2_ for 4 hours. Following this, media was replaced by maintenance media and returned to the incubator. The media was changed every 48 hours until the cells had been in culture for 7 days. The day before the experiments the cells were change to serum-free media (DMEM -Gibco 11966- supplemented with 10 mM Galactose and 1 mM Na-Pyr)

**Intracellular calcium and contraction analysis on hiPSC-CMs using CellOPTIQ® platform**

Both calcium transients and contractility were measured in the optical platform CellOPTIQ® (Clyde Biosciences Ltd., Glasgow, UK), equipped with a stage incubator to allow temperature, CO_2_ and humidity control. Both parameters were measured from a defined area of 200×200 µm in independent wells and the beating rate of the cells was kept to 1 Hz using carbon electrodes. The recordings were made before (baseline) and 15 min after adding Ang-(1-9) or vehicle control. The data are plotted as percentage change of baseline and compared to vehicle control. To perform the calcium measurements, the cells were loaded with 3 µM Fura-4F-AM in serum-free media (30 min, 37°C). Fura-4F-AM is excited intermittently with two LED at 360 and 380nm and the emitted fluorescence at wavelengths <580nm is registered by one photomultiplier at a sampling rate of 2.5 KHz for each channel. The calcium transients are obtained from the ratio 360/380. The analysis of calcium transients was done off-line using CellOPTIQ® software. Calcium transient amplitude, upstroke and rate of decline are the main parameters obtained. The analysis of contraction was made using a high resolution camera (Orca Flash 4.0, Hamamatsu) coupled to CellOPTIQ® platform. A define area of 512×512 pixels is recorded before and after the treatment for 10 sec. at a sampling rate of 0.1 KHz. The videos were analysed off-line applying a proprietary Cell Motion algorithm able to register the contractility of the cells. The information provided includes the contraction amplitude, a surrogate measurement of contraction force, and contraction and relaxation times.

**Statistical analysis**

Data are represented as mean values ± the standard error of the mean (SEM). Paired student’s T-test was performed for direct comparisons and one-way ANOVA with Tukey’s post-test was performed for multiple comparison, with *P*<0.05 considered statistically significant. Data for echocardiography was analysed using repeated measures ANOVA with Tukey’s post-test. A *P* value <0.05 was considered statistically significant. All statistical analysis was carried out using Graphpad Prism 4 software (GraphPad Software Inc., California, USA).

**References**

1. Flores-Munoz M, Godinho BM, Almalik A, Nicklin SA. Adenoviral delivery of angiotensin-(1-7) or angiotensin-(1-9) inhibits cardiomyocyte hypertrophy via the mas or angiotensin type 2 receptor. PLoS One 2012;7:e45564.

2. Gao G, Vandenberghe LH, Alvira MR et al. Clades of adeno-associated viruses are widely disseminated in human tissues. J Virol 2004;78:6381-8.

3. Mano M, Ippodrino R, Zentilin L, Zacchigna S, Giacca M. Genome-wide RNAi screening identifies host restriction factors critical for in vivo AAV transduction. Proc Natl Acad Sci USA 2015;112:11276-81.

4. Ayuso E, Mingozzi F, Montane J et al. High AAV vector purity results in serotype- and tissue-independent enhancement of transduction efficiency. Gene Ther 2010;17:503-10.

5. Inagaki K, Fuess S, Storm TA et al. Robust systemic transduction with AAV9 vectors in mice: efficient global cardiac gene transfer superior to that of AAV8. Mol Ther 2006;14:45-53.

6. Flores-Munoz M, Work LM, Douglas K et al. Angiotensin-(1-9) attenuates cardiac fibrosis via the angiotensin type 2 receptor. Hypertension 2012;59:300-307.

7. Elliott EB, Hasumi H, Otani N et al. K201 (JTV-519) alters the spatiotemporal properties of diastolic Ca(2+) release and the associated diastolic contraction during beta-adrenergic stimulation in rat ventricular cardiomyocytes. Basic Res Cardiol 2011;106:1009-22.

8. Elliott EB, Kelly A, Smith GL, Loughrey CM. Isolated rabbit working heart function during progressive inhibition of myocardial SERCA activity. Circ Res 2012;110:1618-27.

9. Bell RM, Mocanu MM, Yellon DM. Retrograde heart perfusion: the Langendorff technique of isolated heart perfusion. J Mol Cell Cardiol 2011;50:940-50.


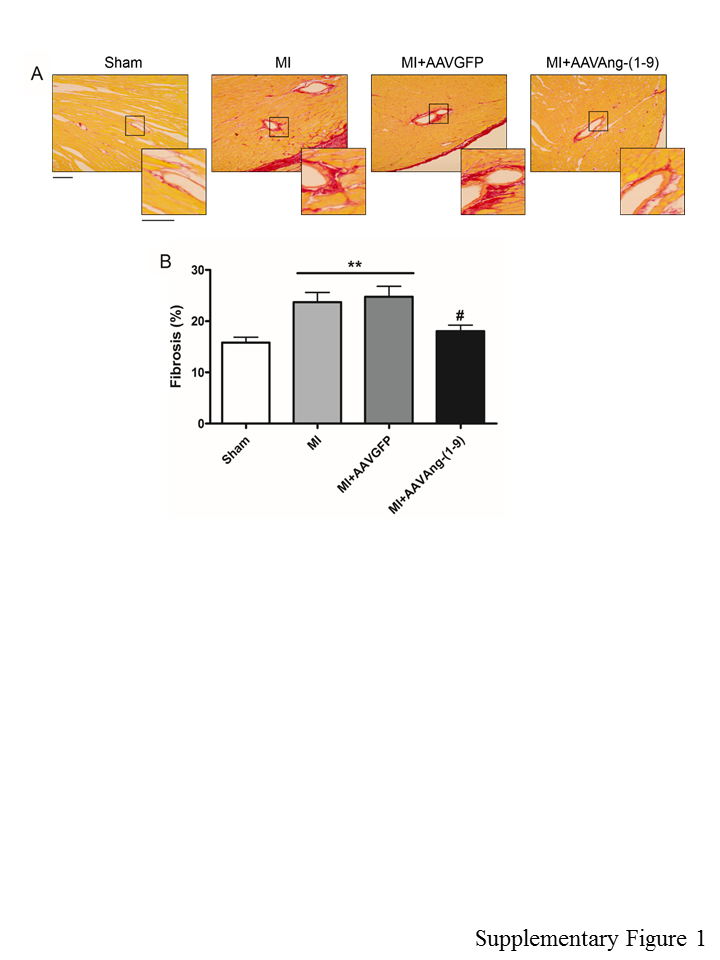


**Online Figure 1. Assessment of cardiac perivascular fibrosis.** (A) Representative images of left-ventricular localised vessels and associated picrosirius red staining of heart sections for each animal group. Magnification= 20x, scale= 200 µm. Zoom insert images scale= 100 µm. (B) Total perivascular cardiac fibrosis quantification for sham, MI, MI/AAVGFP and MI/AAVAng-(1-9) hearts. **= *P*<0.01 *vs.* sham; ^#^= *P*<0.05 *vs.* MI and MI/AAVGFP. *n*= 10, 10, 9 and 8 for sham, MI, MI/AAVGFP and MI/AAVAng-(1-9), respectively. Data presented as mean± SEM.


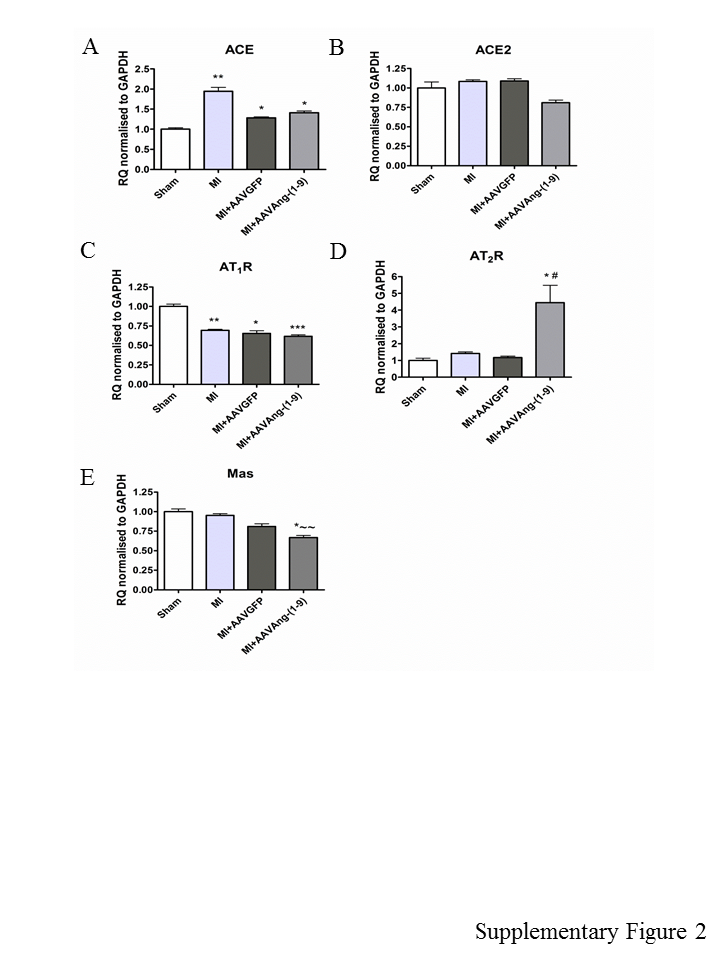


**Online Figure 2. Quantification of RAS components by quantitative RT-PCR.** QRT-PCR-determined gene expression quantification of (A) ACE, (B) ACE2, (C) AT_1_R, (D) AT_2_R and (E) Mas in the hearts of sham, MI, MI/AAVGFP and MI/AAVAng-(1-9) animals. *= *P*<0.05, **= *P*<0.01, ***= *P*<0.001 *vs.* sham, ^#^= *P*<0.05 *vs* MI and MI/AAVGFP, ^~~^= *P*<0.01 *vs.* MI only. *N*= 3 per group. Data presented as RQ± rqmax. Normalisation of expression to a housekeeper (GAPDH) was performed for all samples. MI gene expression was normalised to sham expression, therefore sham expression was arbitrarily set at a RQ= 1. RQ= relative quantification.


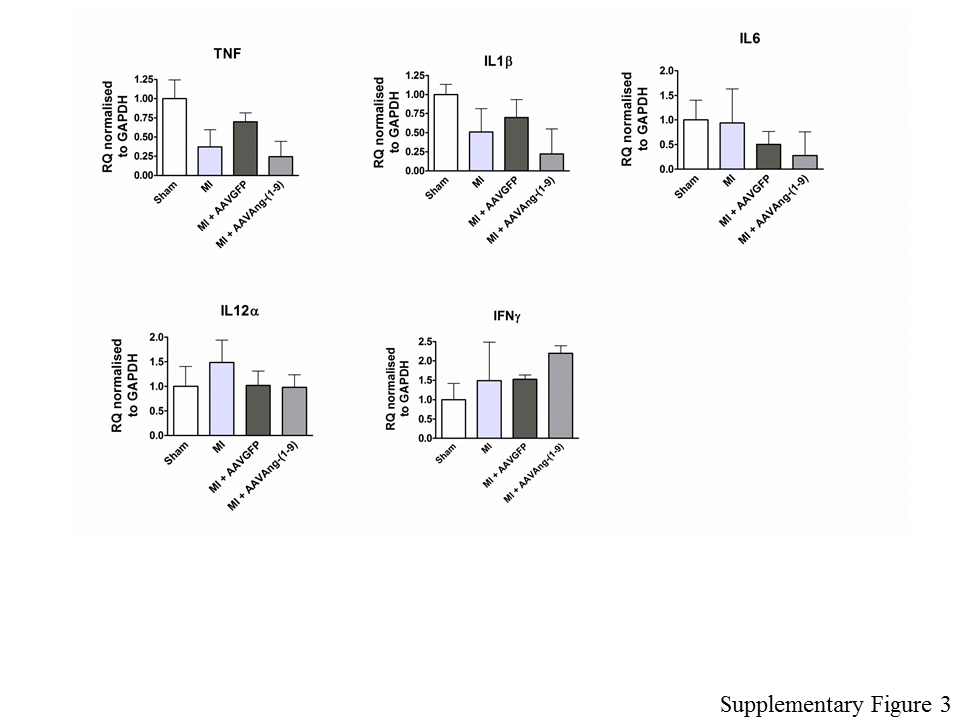


**Online Figure 3. Quantification of inflammatory gene expression by quantitative RT-PCR.** QRT-PCR was used to quantify tumour necrosis factor alpha (TNFα), interleukin (IL) 1β, IL6, IL12α and interferon γ (IFNγ) gene expression in the hearts of sham, MI, MI/AAVGFP and MI/AAVAng-(1-9) animals. *N*= 3 per group. Data presented as RQ± rqmax. Normalisation of expression to a housekeeper (GAPDH) was performed for all samples. MI gene expression was normalised to sham expression, therefore sham expression was arbitrarily set at a RQ= 1. RQ= relative quantification.


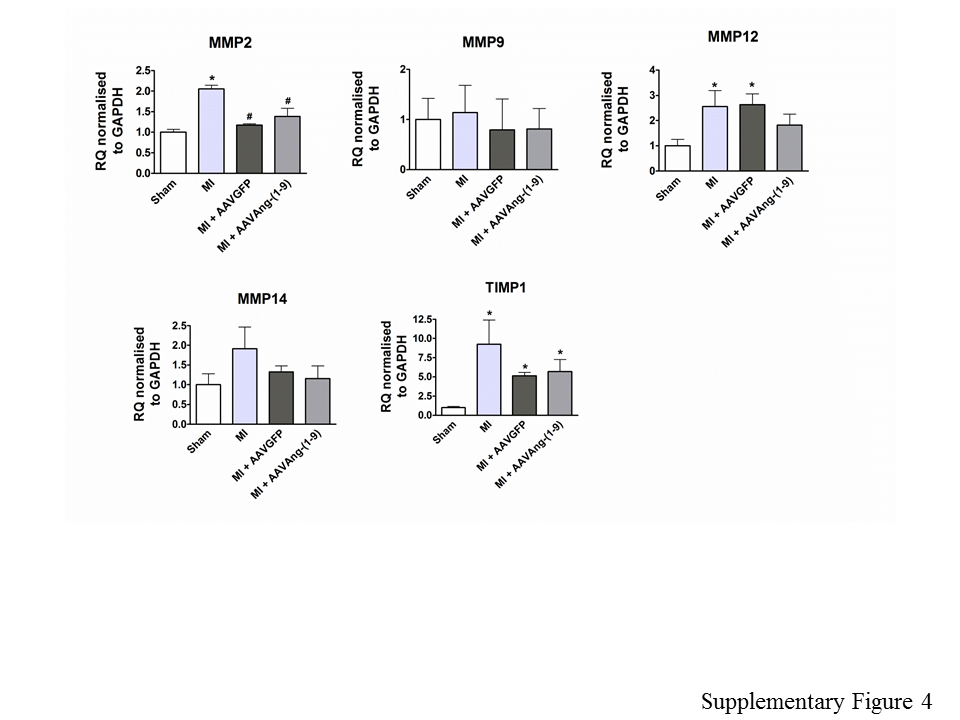


**Online Figure 4. Quantification of matrix metalloproteinase gene expression by quantitative RT-PCR.** QRT-PCR was used to quantify matrix metalloproteinase (MMP)-2, -9, -12, -14 and tissue inhibitor of metalloproteinase (TIMP)-1 gene expression in the hearts of sham, MI, MI/AAVGFP and MI/AAVAng-(1-9) animals. *= *P*<0.05 *vs.* sham, ^#^ = *P*<0.05 vs. MI. *N*= 3 per group. Data presented as RQ± rqmax. Normalisation of expression to a housekeeper (GAPDH) was performed for all samples. MI gene expression was normalised to sham expression, therefore sham expression was arbitrarily set at a RQ= 1. RQ= relative quantification.


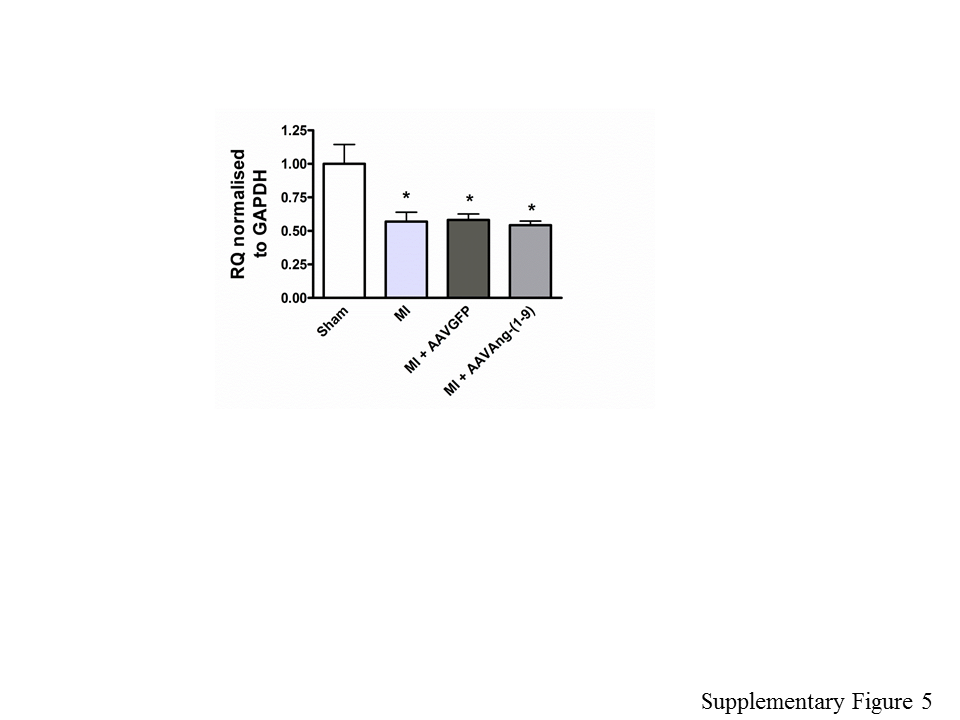


**Online Figure 5. Quantification of SERCA2A gene expression by quantitative RT-PCR.** QRT-PCR was used to quantify SERCA2A gene expression in the hearts of sham, MI, MI/AAVGFP and MI/AAVAng-(1-9) animals. *= *P*<0.05 *vs.* sham. *N*= 3 per group. Data presented as RQ± rqmax. Normalisation of expression to a housekeeper (GAPDH) was performed for all samples. MI gene expression was normalised to sham expression, therefore sham expression was arbitrarily set at a RQ= 1. RQ= relative quantification.


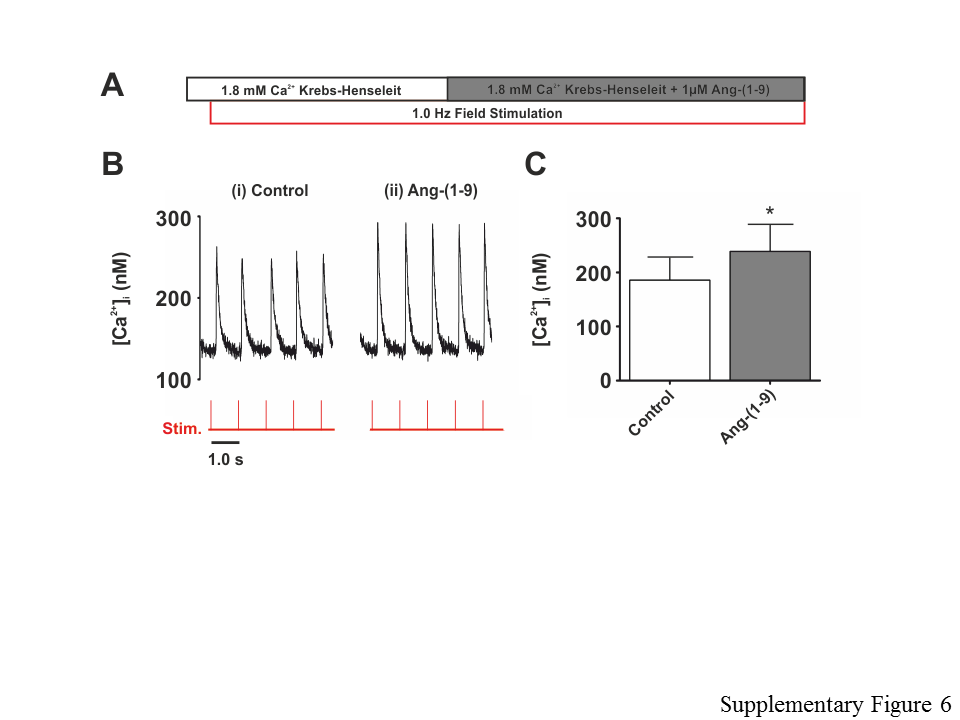


**Online Figure 6. Assessment of excitation contraction coupling in cardiomyocytes isolated from mice subjected to MI.** (A) Perfusion protocol. (B) Example of Ca^2+^-transient traces from cardiomyocytes perfused in (i) 1.8 mM [Ca^2+^]_i_ Krebs-Henseleit followed by (ii) 1 µM Ang-(1-9). Red trace indicates 1 Hz stimulation. (C) Average Ca^2+^-transient amplitude for cardiomyocytes in Krebs-Henseleit and 1 µM Ang-(1-9) (n=9 cells from 3 hearts). *P<0.05 vs. control.


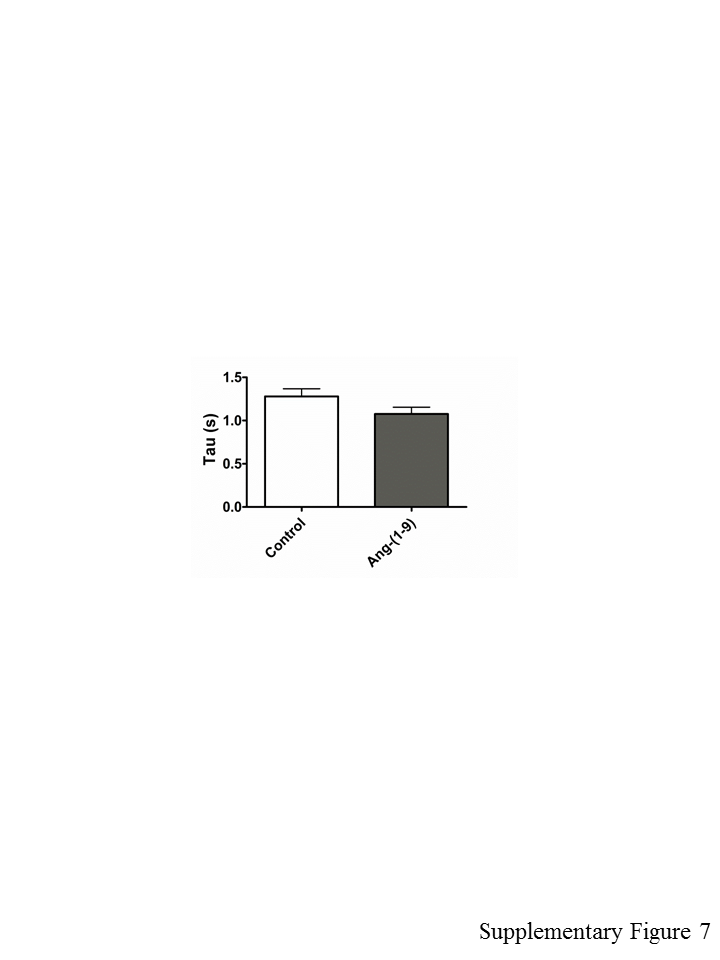


**Online Figure 7. Effects on caffeine induced tau.** (A) Measurements of Tau in untreated (control; N=28) and Ang-(1-9) pre-treated (N=30) myocytes. Data presented as mean± SEM.
